# Integration of healthy volunteers in early phase clinical trials with immuno-oncological compounds

**DOI:** 10.3389/fonc.2022.954806

**Published:** 2022-08-29

**Authors:** Igor Radanovic, Naomi Klarenbeek, Robert Rissmann, Geert Jan Groeneveld, Emilie M. J. van Brummelen, Matthijs Moerland, Jacobus J. Bosch

**Affiliations:** ^1^ Centre for Human Drug Research, Leiden, Netherlands; ^2^ Leiden University Medical Center, Leiden, Netherlands; ^3^ Division of BioTherapeutics, Leiden Academic Centre for Drug Research, Leiden University, Leiden, Netherlands

**Keywords:** phase I, oncology, immunotherapy, healthy volunteers, pharmacology, clinical trials

## Abstract

**Aim:**

Traditionally, early phase clinical trials in oncology have been performed in patients based on safety risk-benefit assessment. Therapeutic transition to immuno-oncology may open new opportunities for studies in healthy volunteers, which are conducted faster and are less susceptible to confounders. Aim of this study was to investigate to what extent this approach is utilized and whether pharmacodynamic endpoints are evaluated in these early phase trials. We conducted a comprehensive review of clinical trials with healthy volunteers using immunotherapies potentially relevant for oncology.

**Methods:**

Literature searches according to PRISMA guidelines and after registration in PROSPERO were conducted in PubMed, Embase, Web of Science and Cochrane databases with the cut-off date 20 October 2020, using search terms of relevant targets in immuno-oncology. Articles describing clinical trials with immunotherapeutics in healthy volunteers with a mechanism relevant for oncology were included. “Immunotherapeutic” was defined as compounds exhibiting effects through immunological targets. Data including study design and endpoints were extracted, with specific attention to pharmacodynamic endpoints and safety.

**Results:**

In total, we found 38 relevant immunotherapeutic compounds tested in HVs, with 86% of studies investigating safety, 82% investigating the pharmacokinetics (PK) and 57% including at least one pharmacodynamic (PD) endpoint. Most of the observed adverse events (AEs) were Grade 1 and 2, consisting mostly of gastrointestinal, cutaneous and flu-like symptoms. Severe AEs were leukopenia, asthenia, syncope, headache, flu-like reaction and liver enzymes increase. PD endpoints investigated comprised of cytokines, immune and inflammatory biomarkers, cell counts, phenotyping circulating immune cells and *ex vivo* challenge assays.

**Discussion:**

Healthy volunteer studies with immuno-oncology compounds have been performed, although not to a large extent. The integration of healthy volunteers in well-designed proof-of-mechanism oriented drug development programs has advantages and could be pursued more in the future, since integrative clinical trial protocols may facilitate early dose selection and prevent cancer patients to be exposed to non-therapeutic dosing regimens.

**Systematic Review Registration:**

https://www.crd.york.ac.uk/prospero/display_record.php?RecordID=210861, identifier CRD42020210861

## Introduction

The field of oncology is rapidly changing, with a major shift from broad-acting cytotoxic chemotherapy to drugs targeting specific molecular and immunological mechanisms (1–4). This is reflected by an ongoing increase in number of immuno-oncological agents in development, even during the COVID-19 pandemic (5). Where traditionally early phase clinical trials with oncological drugs were designed to find a maximum tolerated dose, today’s oncological drugs require a clinical development program based on pharmacologically active dose (PAD) or minimal anticipated biological effect level (MABEL), preferably guided by monitoring of the pharmacological activity (6). Since these drugs have a well-defined molecular target, target engagement and functional downstream effects can be quantified by state-of-the-art molecular and cellular techniques (7). Such an approach enables the evaluation of the relationship between pharmacokinetic (PK) and pharmacodynamic (PD) effects, and the selection of the biologically active dose for subsequent studies. Ideally, this is already done at the earliest clinical stages of drug development, in healthy volunteers (HVs) (8).

Traditionally, early phase clinical trials with non-specific oncological compounds were performed in patients (9). The mechanism of action of these broad-acting cytotoxic compounds did not support evaluation of drug effects in HVs for the obvious reason that the benefit-risk ratio was not acceptable. However, for (certain members of) the new class of targeted immunotherapies pharmacological activity can be evaluated in HVs (9–11). An initial pharmacological evaluation of a novel immuno-modulatory drug in HVs rather than in cancer patients avoids interference of concomitant medication, altered immune status or co-morbidities. Identification of the pharmacologically active dose in HVs would facilitate initial patient studies at selected dose levels and regimens that may translate into clinically desired effects. As such, complicated, inefficient, and time-consuming dose-finding studies in cancer patients could be avoided.

Of course, the benefit-risk assessment for certain immunomodulatory oncology drugs could be negative for HVs. Checkpoint inhibitors, for example CTLA-4 and PD-1 blockers, release the brakes that block the action of the immune system against the tumor. Unfortunately, these compounds also bear the risk for development of immune-related adverse events such as dermatologic, gastrointestinal, endocrine, or hepatic autoimmune reactions. Therefore, this class of compounds is commonly not evaluated in HVs. An alternative approach to enhance the action of the adaptive immune system against malignancies is *via* targeted stimulation of components of the innate immune system, since a fully functional antigen-specific response is dependent on efficient support by innate immune cells and cytokines. This can be reached by specific challenges of innate immune receptors and pathways, for example *via* interleukin receptors or toll-like receptors (TLRs). Whereas checkpoint inhibition theoretically may lead to wide-spread inflammation, targeted stimulation of specific innate immune pathways may result in desirable and well-controllable immune enhancement, which could be evaluated in a safe manner in HVs. We decided to review early phase clinical pharmacology studies with immunomodulatory compounds for oncological conditions addressing the following specific questions: which drug classes have been studied in HVs, did these studies only evaluate safety/tolerability and pharmacokinetics, or also pharmacodynamics, and if so, which type of biomarkers were used to evaluate the pharmacological activity. As a starting point, we selected relevant modes of action based on previously published literature (1, 2), and using the Landscape of Immuno-Oncology Drug Development tool (12).

## Methods

We limited our evaluation to oncological compounds with an immunomodulatory mode of action, defined as modulation of a molecular/cellular immunological target. Relevant modes of action/targets were selected based on the recent drug overviews (1, 2), and by using the Landscape of Immuno-Oncology Drug Development tool (version 2020) (12). Drug targets selected are presented in **Table 1**, grouped by mechanism.

**Table 1:** Overview of the relevant oncology search targets, with their location of expression and intended effect of pharmacotherapy.

Mode of action in oncology	Target	Location of expression	Intended effect of pharmacotherapy
*B cell function or proliferation*			
	CD19	B lymphocytes	Antagonistic
	CD22	Mature B lymphocytes	Antagonistic
	BCMA	Mature B lymphocytes	Antagonistic
*Chemotaxis*			
	H4	Broad expression on immune cells	Agonistic
	CXCR4	Broad expression	Antagonistic
	CCL2/CCR2	Multiple cell types, monocytes, DCs, endothelial cells	Antagonistic
*Immune checkpoint*			
	CD73	Broad expression	Antagonistic
	CTLA-4	Almost exclusively on CD4+ and CD8+ T cells	Antagonistic
	CD27	Naive and effector T cells, NK and B cells	Agonistic
	IDO	Broad expression	Antagonistic
	A2AR	Broad expression	Antagonistic
	Adenosine	Broad availability	Antagonistic
	B7 family (H3)	Broad expression	Antagonistic
	H5 VISTA	Tumor infiltrating lymphocytes, Tregs	Antagonistic
	KIR	NK cells	Antagonistic
	LAG3	Activated T cells, NK cells, Tregs	Antagonistic
	PD-1	Activated T cells, B cells, macrophages	Antagonistic
	PD-L1	Immune cells, especially macrophages and dendritic cells	Antagonistic
	TIGIT	T cells, NK cells	Antagonistic
	TIM-3	Multiple immune cell types	Antagonistic
	ICOS	Activated CD4 and CD8 T cells	Agonistic
	4-1BB	Mainly activated CD4 and CD8 T cells	Agonistic
	GITR	Mainly effector and regulatory T cells	Agonistic
	OX40	Broad expression	Agonistic
*Innate immune response*			
	Dectin	Macrophages, neutrophils, and dendritic cells (DCs)	Agonistic
	EP4 (PGE2)	Broad expression; tumor cells, fibroblasts, and immune cells in tumor stroma	Antagonistic
	IFNαR	Broad expression	Agonistic
	IL12R	T-cells, B-cells, monocytes	Agonistic
	IL8R (CXCR1/CXCR2)	Neutrophils, endothel, myeloid-derived suppressor cells	Antagonistic
	NLRP3	APCs, predominantly macrophages	Unclear
	NOD2	Broad expression	Agonistic
	TLR3	Mainly macrophages, dendritic cells	Agonistic
	TLR4	Myeloid cells	Agonistic
	TLR7	Mainly B cells, monocytes, pDCs	Agonistic
	STING	Broad expression	Agonistic
*Regulation*			
- *activity of immunomodulatory drugs*	CRBN (cereblon)	Broad expression	Agonistic
- *angiogenesis*	VEGF-a/VEGF receptors	Endothelial cells	Antagonistic
- *cell proliferation*	CSF1R	Broad expression	Antagonistic
	CD123 (IL3Rα)	Pluripotent progenitor cells	Antagonistic
- *epidermal growth*	HER1/EGFR	Broad expression	Antagonistic
- *immune cell activity*	CCR5	Mostly T cells, macrophages, DCs, eosinophils	Antagonistic
	CD47	Broad expression	Antagonistic
- *myeloid cell activity*	CD200	Broad expression	Antagonistic
- *phagocytosis*	CD33	Broad expression on myeloid cells	Antagonistic
*T cell function or proliferation*			
	IL-2R	Effector T cells, Tregs	Agonistic (high dose)
	CD3	T cells	Agonistic
	CD38	Plasma B cells, NK cells, B and T cells, other	Antagonistic
	CD40/CD40L	Broad expression (mainly APCs)	Agonistic
*Tumor-associated antigens*			
	CEA	Broad expression	Antagonistic
	FLT3	Hematopoietic progenitor cells	Antagonistic
	MAGE	Mostly tumor-specific	Antagonistic
	HER2	Tumor-specific overexpression	Antagonistic
	EpCam	Epithelial tissues/tumor overexpression	Antagonistic
	GD2	Tumor-specific	Antagonistic
	Mesothelin	Mostly tumor-specific	Antagonistic
	PSMA	Mostly tumor-specific	Antagonistic
*Tumor cell migration, tumor microenvironment*			
	TGFβ	Broad expression	Antagonistic
	CD155	Broad expression	Antagonistic
*Tumor cell survival*			
	AXL	Broad expression	Antagonistic
	JAK1	Broad expression	Antagonistic
	JAK2	Broad expression	Antagonistic
	STAT3	Broad expression	Antagonistic

Targets are based on Tang et al. (1, 2) and Landscape of Immuno-Oncology Drug Development database (12) and were grouped by mode of action in oncology.

### Search strategy

We conducted a comprehensive, electronic search to identify articles indexed in PubMed, Embase, Web of Science and Cochrane Library. The protocol was registered in the international register of systematic reviews (PROSPERO), in accordance with PRISMA guidelines (PROSPERO CRD42020210861) (13). Studies up to 20 October 2020 were extracted. We searched for “healthy volunteers”, “healthy subjects” and at least one of the drug targets as presented in **Table 1**, or alternative synonyms in titles and abstracts. Targets were grouped by their mode of action in oncology. Inclusion criteria were: 1) articles reporting the results of at least one clinical trial; 2) clinical trials conducted in healthy volunteers; 3) articles reporting the clinical evaluation of an immunotherapeutic agent, and the immunotherapeutic agent had a mode of action relevant for an oncological indication (considered relevant if confirmed by a journal publication, in which the possibility of the target in question was investigated or hypothesized), and 4) articles in English. Exclusion criteria were: 1) (systematic) reviews and metanalyses, or population PK studies; 2) articles reporting the results of studies in patients; 3) articles reporting the clinical evaluation of therapies not primarily acting through modulation of the immune system (e.g., tyrosine kinase inhibitor or antibodies such as trastuzumab; 4) articles without full-text availability. Although studies in HVs are primarily conducted during early phase (phase 1a) clinical research, we did not limit our search to only such studies, in order to conduct a more comprehensive review of the literature.

### Data extraction

Relevant data were extracted from the included studies, including treatment, target, study design, study objectives, pharmacodynamic endpoints, number of enrolled subjects, safety/adverse events. Data were grouped and summarized per therapeutic category.

## Results

### Literature search

**Figure 1:**
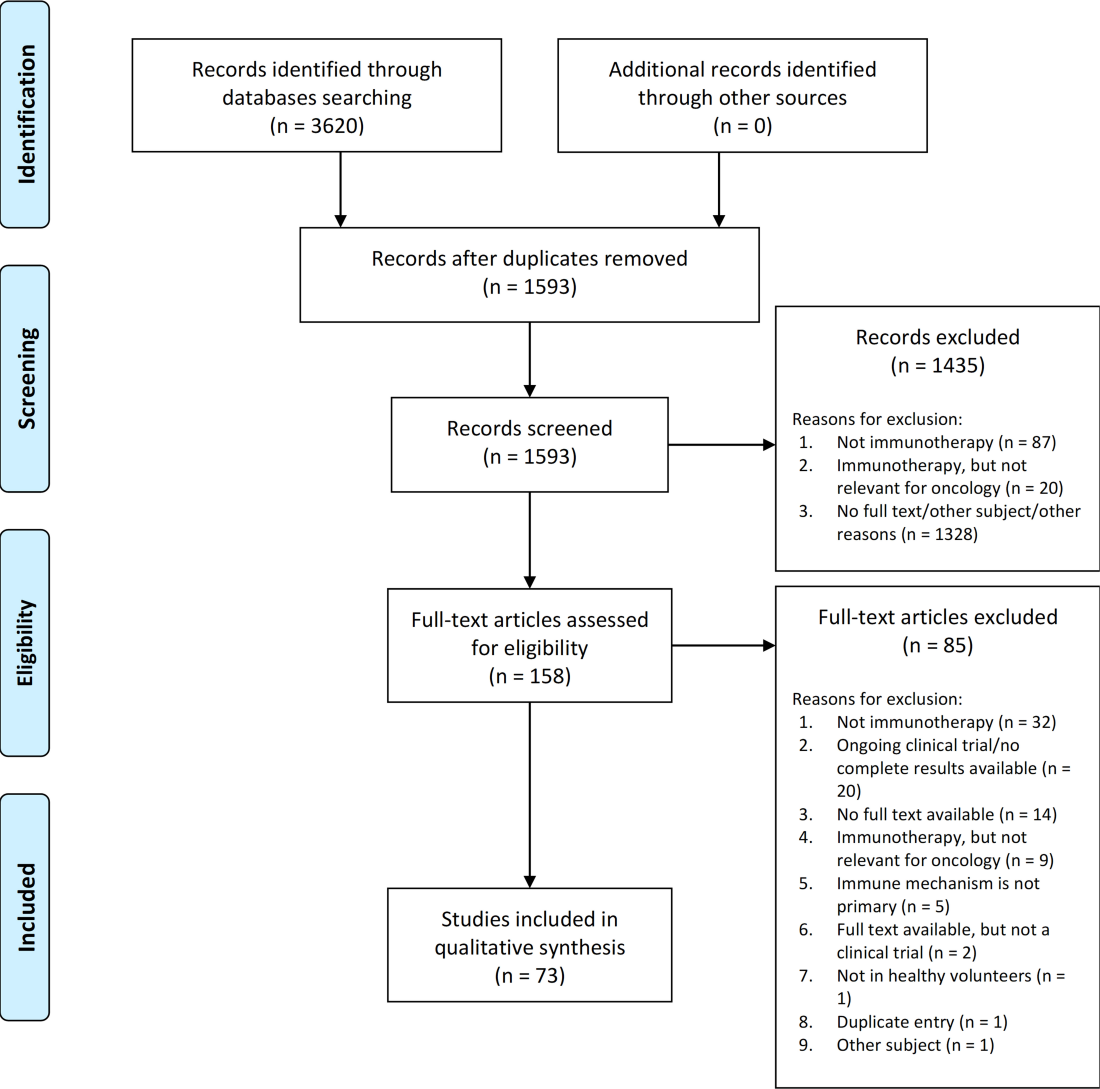
PRISMA diagram showing the total number of studies found by the search, screened, excluded (with reasons for exclusion at screening and full-text review) and included.

A total of 1593 unique entries were identified. Out of those, 158 articles passed the screening and were included for a full-text review. Finally, 73 articles fulfilled the inclusion/exclusion criteria and were included in the review. **Figure 1** shows the PRISMA flow diagram with number of articles in each stage and reasons for exclusion.

### Compounds tested in healthy volunteers

A total of 38 different relevant compounds were evaluated in HV studies in 2352 HVs, based on our search. Studies and compounds are presented in **Table 2**, grouped by target mode of action in oncology and compound’s target/mechanism of action.

**Table 2:** Overview of the included clinical studies conducted in healthy volunteers (HVs) with a compound possibly relevant for immuno-oncology, with their corresponding study design and study endpoints, grouped by potential mode of action in oncology.

Mode of action in oncology	Target/MoA	Compound	Study design	Number of HVs	Study endpoints			Year of publication	Reference
					*Safety*	*PK*	*PD*		
*Chemotaxis*	CXCR4 antagonist	BL-8040	randomized, double-blind, placebo-controlled/open label (2 parts)	33	✓	✓	✓	2017	Abraham et al. (14)
	CXCR4 antagonist	Balixafortide	open label, dose escalation	27	✓	✓	✓	2017	Karpova et al. (15)
	CXCR4 antagonist	Plerixafor	three‐cohort, dose‐escalation, pilot study	21	✓	✓	✓	2011	Lemery et al. (16)
*Immune checkpoint*	Adenonise A_2a_ receptor antagonist	Vipadenant (BIIB014)	prospective, open-label, adaptive, multiple-dose	15		✓	✓	2010	Brooks et al. (17)
	Adenonise A_2a_ receptor antagonist	Istradefylline	single‐center, open‐label, 1‐sequence, 2‐period crossover	20	✓	✓		2018	Mukai et al. (18)
	Adenosine A_2a_/A_2b_ receptor antagonist	AB928	randomized, double-blind, placebo-controlled, SAD and MAD	85	✓	✓	✓	2019	Seitz et al. (19)
*Innate immune response*	CXCR2 antagonist	SCH527123 (navarixin)	randomized, placebo-controlled, crossover	18	✓		✓	2010	Holz et al. (20)
	CXCR2 antagonist	AZD8309	double-blind, placebo-controlled two-way crossover design	20	✓	✓	✓	2013	Leaker et al. (21)
	Dectin receptor agonist	Imprime PGG	SAD	30			✓	2019	Bose et al. (22)
	IFN inducer, TLR3 agonist	Poly(I):poly(C12U)	double-blinded, placebo-controlled, crossover	13	✓		✓	1993	Hendrix et al. (23)
	IFNAR	PEG-IFN α 2a and 2b	randomized, crossover, double-blind, single-dose	16	✓	✓	✓	2010	Garcia-Garcia et al. (24)
	IFNAR	AVI-005 (IFN-α 2b)	open label, single rising dose	28	✓	✓	✓	2007	Patel et al. (25)
	IFNAR	Rh IFNα 2b	randomized, double-blind, two-treatment	24	✓	✓	✓	2000	Rodriguez et al. (26)
	IFNAR	rIFN αA	randomized, placebo-controlled; viral challenge	27	✓		✓	1983	Sarno et al. (27)
	IFNAR	rIFN αA	randomized, placebo-controlled; dose-finding in viral challenge	63	✓		✓	1984	Sarno et al. (28)
	IFNAR	PEG-IFN α	open-label SAD	36		✓	✓	2003	Shiomi, Funaki (29)
	IFNAR	IFNα 2a	double-blind, randomized, two-way crossover	24	✓	✓		1995	Zhi et al. (30)
	IFNAR2B	CIGB-128-A	single-dose	9		✓	✓	2016	Garcia-Garcia et al. (31)
	Oral double prodrug of the TLR7‐specific agonist (RO7011785)	RO7020531	randomized, sponsor‐open, investigator/subject‐blinded, placebo‐controlled, SAD and MAD	70	✓	✓	✓	2020	Luk et al. (32)
	TLR4 agonist	LPS	double-blinded, placebo-controlled, crossover	24	✓		✓	2020	Hijma et al. (33)
	TLR4 agonist	GSK1795091	randomized, double-blind, placebo-controlled	42	✓	✓	✓	2020	Hug et al. (34)
	TLR7 agonist	Imiquimod (with omiganan)	randomized, open‐label, evaluator‐blinded, vehicle‐controlled, parallel‐cohort, dose‐ranging	16	✓		✓	2020	Niemeyer-van der Kolk et al. (35)
	TLR7/TLR8 agonist	Imiquimod	single-dose, placebo-controlled	20			✓	2009	Pasmatzi et al. (36)
	TLR9 receptor agonist	CPG 10101 (ACTILON)	randomized, double-blind, placebo-controlled, dose escalation	48	✓	✓	✓	2007	Vicari et al. (37)
	Type I IFN receptor	IFNβ-1a and IFNβ-1b	single-blind, single-dose, crossover	13	✓		✓	1999	Buraglio et al. (38)
*Regulation – activity of immunomodulatory drugs*	Cereblon (CRBN) modulation	Lenalidomide	randomized, single dose, crossover; study to determine effect on QTc interval	60	✓	✓	✓	2013	Chen et al. (39)
	CRBN modulation	Lenalidomide	open-label, single-center, single dose; study to determine disposition of radioactively labeled lenalidomide	6	✓	✓		2012	Chen et al. (40)
	CRBN modulation	Lenalidomide	open-label, single-center, multiple dose; study to determine distribution of lenalidomide in human semen	24	✓	✓		2010	Chen et al. (41)
	CRBN modulation	Lenalidomide	(1) randomized, single-blind, alternating group, SAD, (2) a randomized, two-way crossover FE (3), a randomized, double-blind, two-group, within-subject, SAD; PK studies (dose proportionality, FE, racial sensitivity)	58	✓	✓		2012	Chen et al. (42)
	CRBN modulation	Lenalidomide	two phase I, crossover studies; DDI studies	50	✓	✓		2014	Chen et al. (43)
	CRBN modulation	Pomalidomide	single center, open-label, non-randomized, 2-part phase 1; DDI study	32	✓	✓		2015	Kasserra et al. (44)
	CRBN modulation	Pomalidomide	phase 1, randomized, double-blind, placebo-controlled; study to determine distribution of pomalidomide in human semen	33	✓	✓		2018	Li et al. (45)
	CRBN modulation	Pomalidomide	2 separate phase 1 open-label, single-dose studies; DDI study	43	✓	✓		2018	Li et al. (46)
	CRBN modulation	Pomalidomide	open-label, randomized, three-period, two-sequence crossover; bioequivalence study	28	✓	✓		2018	Li et al. (47)
	CRBN modulation	Pomalidomide	phase 1, single-center, randomized, crossover; study to determine effect on QTc interval	72	✓	✓	✓	2016	Mondal et al. (48)
	CRBN modulation	Thalidomide	open-label, single-dose; study to determine effects on WBC	2			✓	1992	Neubert et al. (49)
	CRBN modulation	Thalidomide	open label, single dose, randomized, three-way crossover; FE study	13	✓	✓		2000	Teo et al. (50)
	CRBN modulation	Thalidomide	open-label, single-dose, three-way crossover; PK study	15	✓	✓		2001	Teo et al. (51)
	CRBN modulation	Thalidomide	open-label, single-dose, three-way, crossover; bioequivalence study	17	✓	✓		1999	Teo et al. (52)
*Regulation – angiogenesis*	IL-3 receptor	rhIL-3	parallel-group, open-label	19	✓	✓	✓	1997	Huhn et al. (53)
*Regulation – immune cell activity*	CCR5 antagonist	Aplaviroc	open-label, two-part study	32	✓	✓		2008	Adkison et al. (54)
	CCR5 antagonist	Maraviroc	double-blind, placebo-controlled (3 studies); phase 1 studies to assess PK and safety	132	✓	✓		2008	Abel et al. (55)
	CCR5 antagonist	Maraviroc	double-blind, placebo-controlled, crossover (3 studies); DDI studies	39	✓	✓		2008	Abel et al. (56)
	CCR5 antagonist	Maraviroc	open, randomized, placebo-controlled (4 studies); DDI studies	80	✓	✓		2008	Abel et al. (57)
	CCR5 antagonist	Maraviroc	open, randomized, placebo-controlled, crossover (2 studies); DDI studies	28	✓	✓		2008	Abel et al. (58)
	CCR5 antagonist	Maraviroc	open-label/combined double-blind and open-label (2 studies); PK study using radioactively labeled maraviroc	23	✓	✓		2008	Abel et al. (59)
	CCR5 antagonist	Maraviroc	open, randomized, placebo-controlled (2 studies); DDI studies	72	✓	✓		2008	Abel et al. (60)
	CCR5 antagonist	Maraviroc	single-dose, placebo- and active-controlled, five-way crossover; study to determine the effect on QTc interval	61	✓	✓		2008	Davis et al. (61)
	CCR5 antagonist	Maraviroc	open-label, single-dose; study to investigate CYP3A5 genotype on PK	24		✓		2014	Lu et al. (62)
	CCR5 antagonist	Maraviroc	open-label, randomized, crossover (two studies); DDI studies	32	✓	✓		2012	Vourvahis et al. (63)
	CCR5 antagonist	Maraviroc	two studies: double‐blind, randomized (1:1:1), comparative, noninferiority; open‐label, parallel‐group, multiple‐dose; pharmacogenetic study	47		✓	✓	2019	Vourvahis et al. (64)
	CCR5 antagonist	Maraviroc	randomized, open-label, fixed-sequence, crossover; DDI study	12	✓	✓		2014	Vourvahis et al. (65)
	CCR5 antagonist	Vicriviroc	randomized, open-label, parallel group; DDI study	27	✓	✓		2011	Kasserra et al. (66)
	CCR5 antagonist	Vicriviroc	two studies (1): randomized, partially blind, parallel-group (2), randomized, third-party-blind, placebo-controlled, parallel-group; study to assess CNS effects and effect on QTc interval	200	✓	✓		2010	O’Mara et al. (67)
*T cell function or proliferation*	Anti-CD38 monoclonal antibody	TAK‐079 (mezagitamab)	randomized, double‐blind, placebo‐controlled, SAD	74	✓	✓	✓	2018	Fedyk et al. (68)
	IL-1 receptor antagonist	Anakinra	double-blinded, placebo-controlled, crossover	23	✓		✓	2015	Hernandez et al. (69)
	IL-10 receptor agonist	rhIL-10	randomized, double-blind	54	✓	✓	✓	1997	Huhn et al. (70)
*Tumor cell migration, TME*	TGF-βR1 Kinase/ALK5 inhibitor	Galunisertib	open-label	6	✓	✓		2017	Cassidy et al. (71)
	P2X7 antagonist	JNJ-54175446	randomized, placebo-controlled, double-blind, multiple ascending dose	64	✓	✓	✓	2020	Recourt et al. (72)
*Tumor cell survival*	TYK2/JAK1 Inhibitor	PF-06700841 (brepocitinib)	randomized, double-blind, placebo-controlled, parallel-group SAD and MAD	54	✓	✓	✓	2018	Banfield et al. (73)
	JAK1/JAK2 inhibitor	Ruxolitinib	open-label, multiple-dose, single-dose; DDI study	31		✓	✓	2012	Shi et al. (74)
	JAK1/JAK2 inhibitor	INCB018424 (ruxolitinib)	double-blind, randomized, placebo-controlled, SAD, MAD; FIH study	23	✓	✓	✓	2011	Shit et al. (75)

If the same compound is investigated in multiple studies, a brief description of study objectives is included under study design. MoA, mechanism of action; PK, pharmacokinetics; PD, pharmacodynamics; FE, food-effect; SAD,single-ascending dose; MAD, multiple-ascending dose; DDI, drug-drug interactions; WBC, white blood cells.

In terms of study endpoints, 86% of studies investigated the safety, 82% investigated the compound pharmacokinetics and 57% included evaluation of the pharmacodynamic endpoints in the study design. A full overview of the study design and endpoints can also be found in **Table 2**.

Most studies investigated compounds acting on the innate immune system (19 studies) (20–38), followed by compounds with immunoregulatory activity, classified into immunomodulatory (cereblon [CRBN] modulators; 14 studies) (39–44, 46–52) and mediators of immune cell functions (CCR5 antagonists; 14 studies) (54–59, 61–63, 65–67, 76). All the other compounds were investigated in only one or two HV studies. Overall, the studies included single doses, single ascending doses (SAD) and multiple ascending doses (MAD). Most studies were randomized controlled trials, although a substantial percentage (29%) of articles described a non-randomized trial.

### Safety and tolerability in healthy volunteer studies

An overview of the safety findings in HV studies is provided in **Table 3**. Most of the observed adverse events (AE) were Grade 1 and 2, which included gastro-intestinal side effects (nausea, diarrhea, vomiting, constipation), flu-like symptoms (headache, fever, malaise) and cutaneous side effects (pruritus, erythema, dry skin).

**Table 3:** Overview of safety findings in healthy volunteer studies of compounds with proposed mode of action for immuno-oncology.

Mode of action in oncology	Safety findings per group	Target/MoA	Compound
*Chemotaxis*	Mostly Grade 1AEs **Two Grade 3 AEs (asthenia, syncope)**	CXCR4 antagonists	BL-8040
			Balixafortide
			Plerixafor
*Immune checkpoint*	Grade 1 and 2 AEs	Adenonise A2a receptor antagonist	Vipadenant (BIIB014)
			Istradefylline
		Adenosine 2a/2b receptor antagonist	AB928
*Innate immune response*	Grade 1 and 2 AEs	CXCR2 antagonist	SCH527123 (navarixin)
		CXCR2 antagonist	AZD8309
	Grade 1 and 2 AEs **One Grade 3 AE (headache)**	Dectin receptor agonist	Imprime PGG
	Fatigue, chills, headache, flu-like syndrome (no grading reported)	IFN inducer, TLR3 agonist	Poly(I):poly(C12U)
	Grade 1 and 2 AEs **One Grade 3 AE (severe leukopenia)**	IFNAR	PEG-IFN α 2a and 2b
	Headache, chills, myalgia, nausea (no grading reported)		AVI-005 (IFN-α 2b)
	Grade 1 and 2 AEs		rhIFNα 2b
			rIFN αA
			IFNα 2a
		IFNAR2B	CIGB-128-A
	Grade 1 and 2 AEs **Three Grade 3 AEs (two incidences of headache, flu symptoms)**	Type I IFN receptor	IFNβ-1a and IFNβ-1b
	Grade 1 and 2 AEs	TLR4 agonist	LPS
	Grade 1 and 2 AEs **Three Grade 3 AEs (increased heart rate, increased ASAT and ALAT)**	TLR4 agonist	GSK1795091
	Grade 1 and 2 AEs	TLR7 agonist	Imiquimod (with omiganan)
		TLR7/TLR8 agonist	Imiquimod
		TLR9 receptor agonist	CPG 10101 (ACTILON)
*Regulation – activity of immunomodulatory drugs*	Grade 1 and 2 AEs	Cereblon (CRBN) modulation	Lenalidomide
			Pomalidomide
			Thalidomide
*Regulation – angiogenesis*	Grade 1 and 2 AEs	IL-3 receptor	rhIL-3
*Regulation – immune cell activity*	Grade 1 and 2 AEs	CCR5 antagonist	Aplaviroc
			Maraviroc
			Vicriviroc
*T cell function or proliferation*	Grade 1 and 2 AEs	Anti-CD38 monoclonal antibody	TAK‐079 (Mezagitamab)
		IL-1 receptor antagonist	Anakinra
		IL-10 receptor agonist	rhIL-10
*Tumor cell migration, TME*	(no adverse events reported)	TGF-βR1 Kinase/ALK5 Inhibitor	Galunisertib
	Grade 1 and 2 AEs	P2X7 antagonist	JNJ-54175446
*Tumor cell survival*	Grade 1 and 2 AEs	TYK2/JAK1 Inhibitor	PF-06700841 (brepocitinib)
	Grade 1 and 2 AEs **One discontinuation on active treatment due to Grade 4 neutropenia (dose-limiting toxicity)**	JAK1/JAK2 inhibitor	Ruxolitinib

Significant safety findings (apart from Grade 1 and 2 AEs) are bolded. MoA: mechanism of action.

Overall, there were no serious adverse events (SAE) which were assessed to be related to the study drug. There was a single case of dose-limiting Grade 4 leukopenia occurring in JAK1/JAK2 inhibitor ruxolitinib (75). Severe AEs were observed in the chemotaxis category (asthenia and syncope with a CXCR4 antagonist) and with compounds eliciting innate immune response (severe headache, flu-like symptoms and leukopenia with interferons; increased heart rate, increased ASAT and ALAT with TLR agonists; severe headache with dectin receptor agonist Imprime PGG). There were no severe adverse events observed in other categories, including immune checkpoint inhibitor, drugs with regulatory/immunomodulatory activity, drugs acting on T cell function or proliferation and drugs with presumed effect on tumor cell migration and tumor microenvironment.

### Pharmacodynamic effect evaluation in healthy volunteer studies

**Figure 2:**
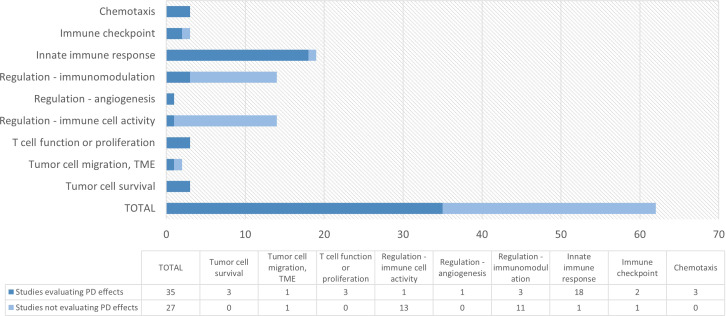
Overview of number of studies with at least one pharmacodynamic (PD) endpoint per target group. Targets included in each category are 1) chemotaxis: CXC4 antagonists; 2) immune checkpoint: A2a and A2a/A2b antagonists; 3) innate immune response: CXCR2 antagonist, dectin receptor agonist, TLR3/4/7/8/9 agonists, IFN; 4) Regulation – immunomodulation: CRBN modulators; 5) Regulation – angiogenesis: IL-3; 6) Regulation – immune cell activity: CCR5 antagonists; 7) T cell function or proliferation: anti-CD38 mAb, IL-1R antagonist, IL-10 agonist; 8) Tumor cell migration: TGF-βR1 Kinase/ALK5 inhibitor, P2X7 antagonist; 9) Tumor cell survival: TYK2/JAK1 inhibitor, JAK1/JAK2 inhibitor; PD, pharmacodynamic; TME: tumor microenvironment.

Pharmacodynamic endpoints evaluated in studies with compounds possibly relevant for immuno-oncology were categorized by mechanism of action and summarized in **Table 4**. In total, there were 27 compounds for which at least one PD endpoint was investigated. All compounds except imiquimod were administered systemically. An overview of the studies evaluating PD endpoints per target group is presented in the earlier discussed **Figure 2**. The majority of HV studies with compounds targeting the innate immune response (consisting of CXCR2 antagonists, dectin receptor antagonist, interferons TLR agonists and P2X7 antagonist) included at least one PD endpoint (18 out of 19 studies) (20–38). Overall, most studies aimed to evaluate the effect of the investigational compound on circulating cytokine/chemokine levels, immune and inflammatory parameters and biomarkers in blood, cell counts, immunophenotype of circulating immune cells, and on the response to an *ex vivo* immune challenge.

**Table 4:** Studies with pharmacodynamic endpoints possibly relevant for oncology.

Mode of action in oncology	Target/MoA[role in immuno-oncology]	Compound(route of administration)	Grouped relevant pharmacodynamic endpoint	Study pharmacodynamic endpoints – detailed
**Chemotaxis**	CXCR4 antagonists [ (77)]	Balixafortid (i.v.)	Phenotyping of circulating immune cells	Complete blood cell count, quantification of CD34+, other immune cells subsets and plasmacytoid dendritic cell progenitors (pro-pDCs)
		BL-8040 (i.v.)		CD34+ and other WBC cell count, expression of CXCR4, surface markers analysis
		Plerixafor (s.c.)		CD34+ cell mobilization; colony forming units (CFU) assay
**Immune checkpoint**	A_2a_R and A_2b_R antagonist [ (78)]	AB928 (p.o.)	Ex vivo challenge assay	pCREB levels in CD8+ cells in whole blood; NECA (adenosine receptor agonist) challenge
	A_2a_R antagonist [ (78)]	Vipadenant (p.o.)	Receptor occupancy	Positron emission tomography (PET)
**Innate immune response**	CXCR2 antagonist [ (79)]	Navarixin (SCH527123) (p.o.)	Cytokine/chemokine levels, immune parameters in blood and cell counts	Sputum neutrophil counts, sputum IL-8 levels, peripheral blood neutrophils
		AZD8309 (p.o.)		Inflammatory cells and mediators in induced sputum and in blood; spirometry
	Dectin receptor agonist [ (22, 80)]	Imprime PGG (i.v.)	Cytokine/chemokine levels, immune parameters in blood and cell counts	Serum IgG and IgM ABA, complete blood counts, circulating immune complex (CIC) levels, complement activity plasma, cytokine and chemokine measurement
	IFNAR [ (81, 82)]	PEG-IFNα 2a and 2b (s.c.)	Cytokine/chemokine levels, immune parameters in blood, phenotyping circulating immune cells	Neopterin and β2-microglobulin (β2M) concentrations in serum, induction of 2’,5’ oligoadenylate synthetase (2’,5’ OAS) mRNA expression, serum IFN antiviral activity
		IFN-β 1a and 1b (s.c.)		PBMC proliferation, CD markers expression, biomarkers (β2-microglobuline, neopterin)
		IFN-α 2b (i.m.)		Neopterin and β2-microglobuline, mRNA expression of the interferon-inducible protein kinase (PKR) and 2’5’ oligoadenylate synthetase (OAS), TNF-α levels
		PEG-IFNα 2a (s.c.)		2’, 5’-OAS levels
	IFNAR/IFNGR [ (81, 83)]	IFN α-2b and IFN-µ (i.m.)	Cytokine/chemokine levels, immune parameters in blood	Serum neopterin, β2-microglobulin (β2M) and 2′–5′ oligoadenylate synthetase (2′–5′ OAS)
	TLR3 agonist [ (82)]	poly(I):poly(C12U) (i.v.)	Phenotyping circulating immune cells; cytokine/chemokine levels in blood	IFN levels, neopterin, T cell subsets, lymphocyte proliferation, NK cell activity
	TLR4 agonist [ (84)]	LPS (i.v.)	Cytokine levels, inflammation parameters, phenotyping of circulating immune cells	Cytokines, cortisol and CRP levels; pain tests
		GSK1795091 (i.v.)		White blood cells count, cytokine levels, leukocyte phenotyping
	TLR7 agonist, double prodrug [ (85, 86)]	RO7020531 (p.o.)	Cytokine/chemokine levels, immune parameters in blood	Cytokine/chemokine levels (IFN‐α, TNF‐α, IL‐12p40, IL‐6, IL‐10, and IP‐10) and neopterin levels
	TLR7/8 agonist [ (85, 87)]	Imiquimod (topical)	Phenotyping circulating immune cells; cytokine levels in blood; immunohistochemistry	Peripheral blood lymphocytes subpopulations, cytokines biomarkers, immunohistochemistry
	TLR9 agonist [ (88)]	CPG 10101 (Actilon) (s.c.)	Peripheral blood count; autoimmune diagnostic biomarkers	Cytokine levels, leukocyte count, ANA, anti-dsDNA and RF
**Regulation – activity of immunomodulatory drugs**	CRBN modulation [ (89, 90)]	Thalidomide (p.o.)	Phenotyping circulating immune cells	White blood cells CD surface markers expression
**Regulation – angiogenesis**	IL-3 agonist [ (91)]	rhIL-3 (s.c.)	Peripheral blood cell counts	Blood cells and CD34+ progenitor cells count
**T cell function and proliferation**	IL-1 receptor antagonist [(92)]	Anakinra (s.c.)	Cytokine levels; cell counts	Cytokine levels, white blood cells count, sputum neutrophils
	IL-10 receptor agonist [(93)]	rhIL-10 (s.c.)	Cytokine levels; cell counts	Cytokine levels; white blood cells and platelet count
	anti-CD38 monoclonal antibody [(94)]	TAK‐079 (mezagitamab) (i.v./s.c.)	Immune cell counts	Plasmablasts and NK cells levels
**Tumor cell migration, TME**	P2X7 antagonist [(95)]	JNJ-54175446 (p.o.)	*In vivo* challenges, ex vivo challenge assay	NeuroCart, PharmacoEEG, dexamphetamine challenge, LPS/BzATP induced IL-1β release assay
**Tumor cell survival**	JAK1/JAK2 inhibitor [(96, 97)]	Ruxolitinib (p.o.)	Ex vivo challenge assay	IL‐6 induced activation of JAK/STAT pathway, levels of phosphorylated STAT3 (pSTAT3)
	TYK2/JAK1 inhibitor [(97)]	Brepocitinib (PF-06700841) (p.o.)	Blood biomarker levels	JAK1 downstream biomarkers (IP-10, hsCRP, neutrophils, lymphocytes)

Studies are grouped by mode of action in oncology, and investigated pharmacodynamic endpoints were grouped by compound mechanism of action. MoA: mechanism of action; i.v.: intravenous; s.c.: subcutaneous; p.o.: peroral; i.m.: intramuscular.

All three studies with anti-chemotaxis agents (CXCR4 antagonists) (14–16) included PD markers, such as the mobilization of immune cell subsets including CD34+ hematopoietic stem cells, and receptor and surface marker expression (i.e., surface markers of mature immune cell subsets such as T, B and NK cells, T cell subpopulations, monocytes and plasmacytoid dendritic cell progenitors). For the immune checkpoint compounds (adenosine antagonists), positron emission tomography (PET) was used to investigate adenosine A_2a_ receptor occupancy (17). In another study target engagement by a double adenosine A_2a_ and A_2b_ receptor antagonist was determined by *ex vivo* challenge with a synthetic adenosine agonist (5’-N-ethylcarboxamidoadenosine; NECA) and subsequent evaluation of the levels of the phosphorylated cyclic AMP (cAMP) response element binding protein (CREB) in CD8+ cells (19).

In the category of compounds affecting the tumor microenvironment (TME), one study was identified investigating a P2X7 antagonist. The compound’s peripheral target engagement was demonstrated by an *ex vivo* immune challenge, evaluating the LPS/BzATP-induced IL-1β release in peripheral blood mononuclear cell (PBMC) cultures (72).

Studies with compounds targeting the tumor cell survival pathways included JAK1/JAK2 and TYK/JAK1 inhibitors. One study measured the levels of phosphorylated STAT3 (pSTAT3) after *ex vivo* cell stimulation with IL-6 (75), whereas in the other study markers downstream from JAK1 were evaluated (circulating IP-10 and hsCRP levels and neutrophil and lymphocyte count) (73).

Finally, of note was the observable lack of pharmacodynamic endpoints in HV studies which investigated the immunomodulatory drug thalidomide (and analogues) and CCR5 antagonist maraviroc (and analogues), where almost all of the studies only assessed the safety and pharmacokinetics of the compounds.

## Discussion

A review of literature on published early phase clinical studies using immuno-oncology compounds in healthy volunteers following PRISMA guidelines and PROSPECT registration was presented in this article. In total, we have found 73 published articles and included 38 different potential immunotherapeutic compounds that have been conducted in HVs.

The majority of the studies investigated immunomodulatory compounds such as interferons, TLR agonists and drugs targeting chemokine receptors. Studies evaluating oncolytic viruses and T-cell based therapies were excluded from our review, since the primary mechanism of action of these compounds is based on an antigen-specific pharmacological activity and not a general immunomodulatory effect. Noteworthy was the lack of studies investigating immune checkpoint inhibitors (other than adenosine antagonists) in HVs, which might be explained by the potential immune-related adverse events of such compounds, typically with a delayed onset and prolonged duration, resulting in an unfavorable benefit/risk ratio for a HV study (98). For comparison, almost all the innate immune system targets mentioned in **Table 1** were investigated in HV studies, while at the same time only one immune checkpoint target was identified.

Thalidomide and analogues were investigated in 14 HV studies (**Table 2**), but only one study included a relevant PD endpoint investigating immunophenotype of circulating immune cells (**Table 4**) (49). Thalidomide is a drug with troublesome history but remarkable revival decades later as an anti-myeloma drug (99), and it has been discovered that thalidomide and its newer analogues lenalidomide and pomalidomide elicit multiple direct and indirect immune-related anti-myeloma effects, among others by modulating the ubiquitin E3 ligase cereblon (CRBN) (89, 100, 101). Although their indirect immunomodulatory properties in multiple myeloma have been clearly demonstrated (102, 103), previous research might have been more focused on their direct anti-tumor mechanism, requiring the drug effects to be investigated mostly in patients. Similarly, the difference is also significant when looking at CCR5 antagonist maraviroc and its analogues, with 14 HV studies in total and no studies investigating relevant PD, since these compounds are developed and approved as anti-HIV drugs, and their importance for immuno-oncology has only recently been uncovered (104).

### Safety perspective

Overall, the adverse event profiles for the compounds evaluated in HVs were acceptable and within the normal range for HV studies, when compared to the available literature. One such published review reported that among 475 phase 1 studies in 27185 HVs, 33% of studies reported at least one severe AE, which is significantly more than what was captured in our review, which was 6 (8%) of the included studies (105). Although we did not directly compare the safety findings in HV studies to the studies with same compounds in patients, safety is expected to be comparable between two populations with regards to drug-related adverse events.

From a safety perspective, drugs targeting proteins that are widely present in healthy tissues inherently carry a higher risk for (auto-immune) toxicity. Safety findings in the identified studies were overall well acceptable, although there were some expected higher-grade toxicities observed in studies with compounds targeting the dectin receptor, CXC4 receptor, JAK1/JAK2 and some specific components of the innate immune pathways. The majority of the severe adverse events of the latter subgroup mainly relate to their inherent ability to boost the (innate) immune response, but also to the immunosuppressive effects of interferon, which can lead to interferon-induced neutropenia (106). Severe neutropenia observed with ruxolitinib has been previously reported (107), which can be explained by the drug’s mechanism of action: its anti-JAK1/JAK2 activity decreases T cell activation and neutrophil activity.

Notably, there were no severe adverse events observed in the immune checkpoint group, where adenosine antagonists were well tolerated up to the highest dose tested, while demonstrating a robust target engagement (19). This points to the possibility of investigating other immune checkpoint modulators in early proof-of-concept clinical trials in HVs. Obviously, a reason to remain cautious is the risk of inducing late-onset immune-related adverse events (irAEs) and autoimmunity in HVs. However, future testing of such compounds in HV trials should not be categorically ruled out, especially when compounds with more controllable immune-mediated mode of actions and favorable immune-related toxicity profiles can be developed.

### Pros and cons of healthy volunteer trials

There are numerous advantages of performing early phase clinical trials in HVs before studies in patients are initiated. This is a relatively homogenous population, void of any confounders such as comorbidities or concomitant medications. Patient pre-selection and strict inclusion criteria in early oncology trials may lead to a selection bias, preventing the extrapolation of the results to a general population (108). Practically, recruiting HVs for early phase trials is easier, faster and less expensive, with significantly lower drop-out rates and better compliance which eventually leads to better data quality. Importantly, a HV-based study including PD endpoints can assist in selecting a pharmacologically active dose for the first phase 1B trial, which avoids inefficient dose finding studies in the target population and inclusion of patients in studies with pharmacologically inactive doses (3). Specifically for immunomodulatory compounds, the comparison of immunocompetent HVs with immunosuppressed cancer patients in an integrative study design may be advantageous. Our review shows that testing selected immuno-oncological compounds in early phase clinical trials integrating HVs is feasible from a safety perspective. Furthermore, based on our findings, relevant PD effects were evaluated in 57% of the identified studies, with studies testing compounds targeting the innate immune system being more likely to include at least one PD endpoint. With lack of efficacy as the primary source of failure in later stage clinical research (109), it is of paramount importance to demonstrate pharmacological activity of a new compound early in clinical development in double-blind randomized controlled trials with clear PD endpoints, prior to moving to the more expensive and significantly lengthier patient trials with clinical endpoints (110).

On the other hand, the critical point-of-attention for evaluation of oncology drugs in HVs is the benefit/risk ratio, with is obviously different between cancer patients and HVs. Moreover, for certain compounds evaluation of effects in HVs is not relevant because of low or absent target expression, which is for example the case for tumor-associated antigens. For the presented classes of immunomodulatory compounds this does not represent a problem: these drugs have targets that are expressed in healthy cells or tissues, and consequently there is a possibility to study drug concentration versus effect in HVs. HV trials evaluating JAK1 tyrosine kinase inhibitors (73–75) or an adenosine receptor antagonist (19) included evaluation of cell-based target engagement. Adenosine has been identified as one of the key immunosuppressive molecules reducing effector immune cell activity in TME, which subsequently led to development of inhibitors of the adenosine pathway (78). An example of a successful early phase program in HVs with a compound targeting an immune checkpoint is that of the double adenosine receptor antagonist AB928 (etrumadenant) which is currently undergoing phase 1b/2 trial in cancer patients (ClinicalTrials.gov identifier: NCT04660812) (111), after PK/PD profiling and efficient dose selection in a phase 1 HV study (19). Challenges to investigating immune checkpoint inhibitors in HVs comes from their biological characteristics – they are mostly constructed as IgG monoclonal antibodies (mAbs). This has an impact on the absorption, distribution and metabolism of these compounds, introducing a significant interindividual variability to the PK profiles. Furthermore, target-mediated drug disposition (TMDD) of the mAbs may be one of the main culprits for the complex PK profiles observed with mAbs, considering the availability of the drug molecular target(s) changes with disease state (or absence of disease). These aspects make it particularly challenging to investigate mAb-based checkpoint blockade in HV trials (112, 113).

Since a drug’s effective concentration depends on the clinical context and the desired extent of activity on the specific cellular pathways in a particular condition (114), the PK/PD relationship assessed in HVs does not necessarily translate 1:1 to the targeted patient population. This may represent a significant challenge for immunotherapeutic compounds, such as CXCR2, CXCR4 and CD38 antagonists.

The main function of the chemokine receptor CXCR2 is to regulate the migration and efflux of neutrophils from the bone marrow and it also plays a role in controlling the migration of myeloid derived suppressor cells (MDSCs) to TME in patients. Increased CXCR2 signaling leads to increased levels of neutrophils and MDSCs in TME, which has been associated with abrogated anti-tumor effects of immunotherapy and poorer clinical outcomes. Depletion of neutrophils and MDSCs by CXCR2 antagonists has been shown to increase the numbers and activity of tumor-infiltrating CD8+ T cells, preventing tumor growth and metastasis (115). Of significance for early phase clinical studies could be the ability to investigate the proof-of-concept of CXCR2 engaging compounds to address targeting of CXCR2 already expressed in immune cells of HVs.

In malignancies, the chemokine receptor CXCR4 has been shown to be overexpressed in various tumor cell populations, causing tumor cell migration, angiogenesis, and tumor progression. Blocking this pathway may therefore be an attractive strategy in tumor immunotherapy (77). CXCR4 antagonists work by disrupting the CXCL12/CXCR4 pathway, thereby inducing the mobilization of stem cells to the periphery, making them valuable in the context of harvesting CD34+ cells from both HVs and patients for hematopoietic stem cell transplantation (14).

CD38 is a glycoprotein overexpressed in certain autoimmune conditions (68), and multiple myeloma, where CD38 antagonism by anti-CD38 mAbs can directly deplete CD38+ myeloma cells (94). Nonetheless, anti-CD38 mAbs have been also shown to successfully deplete the MDSCs and regulatory T cells, thereby reverting the tumor-induced immunosuppression and restoring the anti-myeloma effector T cell functions (94). Such indirect cellular immune mechanisms might already be investigated in the context of proof-of-concept HV trials. Thus, in an integrative clinical study design for immunotherapeutic compounds such as CXCR2, CXCR4 and CD38 antagonists, the variability of target expression in HVs compared to cancer patients should be considered when investigating the PK/PD relationship in HVs for translation into the patient setting.

As outlined in a recent review, there are several additional obstacles that should be taken into account when designing early phase oncology trials in HVs, ranging from more stringent requirements for the pre-clinical pharmacology experiments to alternative study designs, to starting dose selection (below the pharmacologically active dose in HV studies, different than for patients), and maximum exposure (with the difficulty to justify dose escalation above the no observed adverse effects level, NOAEL, in HVs) (116). Obviously, the challenge for future early phase clinical design in oncology will be to further integrate HVs using more sophisticated methodology to measure PD endpoints, and to combine HVs and patients in an integrative clinical trial design.

### Limitations of the study

The findings of this systematic review must be observed in light of some additional considerations. The interpretation of the primary immune-related mechanism of action of a compound is potentially ambiguous. The exclusion of several compounds (listed in **Figure 1**) deserves a separate justification. Although direct tumor-targeting drugs such as trastuzumab, sunitinib and lapatinib were intentionally not included in this review, we are aware that evidence exists that the activity of these and similar compounds may be partly attributed to the activation of the innate and adaptive immune responses, mainly by induction of CD8+ T-cell responses or inhibition of immunosuppressive Treg cells (117, 118). However, they are typically not considered direct immunotherapeutic compounds. Furthermore, calcineurin inhibitor cyclosporine A and protein kinase C inhibitor sotrastaurin, together with vaccines against hepatitis B and human papillomavirus (viruses known to cause malignancies) were not included in immunological targets presented in **Table 1**, even though strictly fulfilling our definition of immunotherapeutic agents (119–122). The first two were not included in the original search due to not (yet) being recognized as relevant targets in in immuno-oncology, meaning the possible use in immuno-oncology was not confirmed by literature, although that might change in the future. Although several HV studies with compounds targeting tumor-associated antigens (TAAs) were identified, we decided to omit those studies, since expression of TAAs in HVs is either absent or low, making the relevance of PD endpoints less obvious in HVs. More specifically, FLT3 tyrosine-kinase inhibitors aimed against acute myeloid leukemia (AML) cells and BCR-ABL-derived peptide vaccine aimed against chronic myeloid leukemia (CML) cells were investigated in HVs (123–126). Importantly, the assessment whether a target could be relevant for oncology was also based on the review by Tang et al. (1, 2) and the Landscape of Immuno-Oncology Drug Development tool (12). Obviously, the clinical relevance as oncological targets remains to be proven for many of them and insights are quickly changing. We did not aim to give a complete overview, but rather an indication of the current state of immuno-oncology drug development studies that integrate HVs in early phase clinical trial protocols.

### Conclusion

In conclusion, the findings of our systematic review show the potential value of HV studies for investigational oncology compounds with an immunomodulatory mechanism of action. For all identified drug classes, the observed safety profiles in HV were favorable, and for many compounds the drug concentration versus activity relationship could be evaluated based on incorporated PD endpoints. As such, the obtained insights can guide selection of a safe and pharmacologically active dose for the phase 1B/2A trial in patients. Based on a thorough benefit/risk assessment, the integration of HVs in early phase drug development programs for immuno-oncological compounds can be considered on a case-by-case basis and may have significant advantages for the later clinical development program.

## Data availability statement

The original contributions presented in the study are included in the article/supplementary material. Further inquiries can be directed to the corresponding author.

## Author contributions

IR, EB, and NK conceived the idea, designed the study protocol and devised the search strategy. IR conducted the screening and full-review of the articles based on systematic search strategy, and extracted data per protocol. EB checked the included articles and extracted data for final decisions. IR wrote the first draft, managed the review process and finalized the manuscript based on co-authors’ feedback. MM, RR, and GG contributed to the review and interpretation of the results. JB supervised the process, edited the manuscript and provided final input. All authors discussed the results and provided feedback to the manuscript. All authors contributed to the article and approved the submitted version.

## Acknowledgments

The authors would like to thank Jan W. Schoones (Leiden University Medical Centre) for help with preparing the final search strategy.

## Conflict of interest

The authors declare that the research was conducted in the absence of any commercial or financial relationships that could be construed as a potential conflict of interest.

## Publisher’s note

All claims expressed in this article are solely those of the authors and do not necessarily represent those of their affiliated organizations, or those of the publisher, the editors and the reviewers. Any product that may be evaluated in this article, or claim that may be made by its manufacturer, is not guaranteed or endorsed by the publisher.
